# Barriers to HPV Vaccination Among Unvaccinated, Haitian American College Women

**DOI:** 10.1089/heq.2017.0028

**Published:** 2018-06-01

**Authors:** Dudith Pierre-Victor, Dionne P. Stephens, Angela Omondi, Rachel Clarke, Naomie Jean-Baptiste, Purnima Madhivanan

**Affiliations:** ^1^ National Cancer Institute, Bethesda, Maryland.; ^2^ Florida International University, Miami, Florida.; ^3^ Barry University School of Podiatric Medicine, Miami Shores, Florida.

**Keywords:** barriers, college women, Haitian-American, HPV vaccination

## Abstract

**Background:** Haitian women residing in the United States are disproportionately affected by cervical cancer. Human papillomavirus (HPV) vaccine has been licensed in the United States since 2006. Vaccination rates are less than optimal overall, and the rates are particularly low among young black women. We investigated barriers to HPV vaccination in a sample of Haitian American college women.

**Methods:** Thirty self-identified Haitian American women, aged 17−26 years, were recruited from a large university campus in southeastern United States (*n*=30). They completed in-depth face-to-face interviews. The research team analyzed the transcripts using thematic analysis.

**Results:** More than half of the participants (*n*=18) had not yet initiated the HPV vaccine series. Most of the unvaccinated participants stated that they had received a provider recommendation for the vaccine. Lack of provider recommendation, negative vaccine perception and attitudes, and side effect concerns constituted barriers to vaccination.

**Conclusions:** Haitian American college women at high risk of cervical cancer have cited several barriers to HPV vaccination, with the most prominent being lack of physician recommendation. Healthcare providers should continue recommending the vaccine to college women as many of them may not have received a recommendation. When recommending the HPV vaccine, discussions should be framed with the intent to positively influence HPV vaccine perceptions and ultimately vaccine attitudes.

## Background

The burden of cervical cancer incidence and mortality among Haitian women is high in their homeland where screening programs are not widely implemented.^1^ The high cervical cancer burden persists among Haitian women residing in the United States.^2,3^ In Little Haiti, a predominantly Haitian community in Miami, Florida, the estimated incidence of cervical cancer was 38.0 per 100,000 compared with 9.1 per 100,000 for the state of Florida for the 2000–2004 period.^4^

Nearly all cervical cancer cases result from high-risk human papillomavirus (HPV) infections.^5^ Generally, the immune system rids the body of HPV infection, and clearance occurs ∼12 to 24 months after acquisition.^6^ Persistent HPV infection results in precancerous lesions; in the absence of timely screening and medical interventions, cervical cancer ensues.^7^

Differences in HPV clearance have been reported in the literature. Banister et al. conducted a study among European and African American females and found that the time from HPV acquisition to clearance was longer among African Americans (601 days) compared with European Americans (316 days).^8^ The authors posited that the longer clearance time is a contributing factor to the cervical cancer disparities observed among African Americans.^8^ Among Haitian women, lack of cervical cancer screening awareness, lack of access to screening services, and negative perceptions of cervical cancer screening, in addition to the longer time to clearance, further increase their risk of developing and dying from cervical cancer.^2,3,9,10^

In 2006, the quadrivalent HPV vaccine (HPV4, Gardasil) was licensed in the United States.^11^ Subsequently, the bivalent vaccine (HPV2, Cervarix)^12^ and the nonavalent vaccine (Gardasil 9)^13^ were licensed in 2009 and 2014, respectively. Any one of the vaccines confers protection against HPV types 16 and 18, which are most commonly associated with oral and anogenital cancers.^5,14^ However, vaccination rates remain low, particularly among groups at higher risk of HPV-related cancers.^15^ Approximately half of the female college population is infected with HPV at any given time.^16^

Although HPV infection is prevalent among college women, their HPV vaccination rates remain far below the Healthy People 2020 goal of 80% coverage.^17^ Only 49% of college women aged 18–26 years have been vaccinated,^18^ with black and Hispanic college women having lower rates compared with their white counterparts.^18–20^ Young Haitian women are at high risk of cervical cancer, but their vaccination rates are low. Although the rates of cervical cancer are higher among Haitians compared with African Americans, a previous study has reported lower HPV vaccination rates among Haitian (31%) compared with African American adolescents (47%).^21^ Lack of HPV knowledge and lack of HPV vaccine awareness among Haitian parents constitute barriers to getting their daughters vaccinated.^21,22^

### Barriers to HPV vaccination

Since the licensure of the vaccine, parents have expressed concerns that their adolescents may interpret receipt of the vaccine as a passport to initiate sexual activity^23,24^ and for young adults to increase risky sexual behaviors.^25,26^ Several factors, including lack of HPV vaccine knowledge, lack of awareness, and lack of provider recommendation, have been identified as potential barriers to HPV vaccination.^27^

Research studies have consistently indicated provider recommendation as a major HPV vaccine facilitator and the lack thereof as a barrier. The lack of provider recommendation has been consistently cited as the reason for young adults to not get the HPV vaccine and for parents to not get their adolescents vaccinated.^21,28–36^ Previous studies have also reported that healthcare providers face barriers to making the HPV vaccine recommendation. Findings revealed that many physicians were concerned about parents' negative attitudes toward vaccines and parents' adverse effect concerns,^37–39^ as well as inadequate insurance coverage and potential reimbursement issues.^38,40^

### The current study

As college women, young Haitian American women are at great risk of HPV acquisition, and they are also at a stage where they may play a major role in HPV vaccine decision-making for themselves. Given the disproportionate burden of cervical cancer among adult Haitian women, understanding factors that facilitate or hinder HPV vaccination in this population is crucial to ensuring optimal vaccination rates and ultimately reducing cervical cancer disparities. Several studies have investigated factors that influence HPV vaccine uptake among college women.^18,19,41,42^ However, studies investigating these factors among ethnic minorities that are disparately affected by cervical cancer are lacking, and these studies can facilitate message tailoring for ethnic minorities.

The present study investigated barriers to HPV vaccination among Haitian American college women. This study is part of a larger qualitative project that explored facilitators of and barriers to HPV vaccination among Haitian American college women to facilitate HPV vaccination in this population at high risk of cervical cancer. Although we interviewed both vaccinated and unvaccinated Haitian American women, this article focuses on the barriers to HPV vaccination among participants who were not yet vaccinated. The role of provider recommendation style in HPV vaccine uptake among Haitian American college women has been published elsewhere.^43^

## Methods

Since our goal was to explore Haitian American college women's barriers to vaccination, we used a qualitative approach.

### Participants

Recruitment for this study took place at a large minority-serving university in the southeastern part of the United States. Thirty participants were recruited through an online psychology research pool known as Sona. Through Sona, students enrolled in psychology courses were screened for eligibility before scheduling their interviews; criteria included self-identifying as Haitian, female, and being between 17 and 26 years of age. We wanted to interview participants who were in the vaccine-eligible age group.

### Procedures

The principal investigator conducted the individual interviews at a time selected by the participants. Study participants underwent a consent process, which included reading the letter of consent approved by the university's Institutional Review Board and asking questions to the interviewer. Participants then completed a demographic questionnaire that collected information about age, ethnic identity, and country of birth. For the few participants who were younger than 18, their parent's consent was obtained before the interview.

A series of semistructured, yet flexible, questions were developed based on relevant research.^21,22,44,45^ Interviews began with focused questions to establish HPV vaccination knowledge and beliefs. Interview questions included the following: *Have you ever heard of human papillomavirus or HPV? Where/how did you hear about it? What do you know about the vaccine? Where/how did you get this information? Who should get the HPV vaccine? Have you initiated the HPV vaccine series? What/who do you think influenced you to not get the vaccine?*

Interviews were conducted in a private conference room between June 2014 and March 2015, with each session lasting between 15 and 65 min. Each session was audio-taped and the interview length varied by participants' level of awareness of HPV and experience with HPV vaccine messages. Since the study population included only college-enrolled Haitian American women, the interviews were conducted in English.

### Data analysis

A thematic analysis was undertaken. Data collection and analysis proceeded simultaneously.^46^ The interviews were transcribed and then verified by one study investigator to ensure completeness, accuracy of the discussion content, and a high quality of transcription. A preliminary coding framework related to HPV vaccination knowledge and attitudes was constructed by two study authors and the principal investigator who conducted the interviews after an in-depth reading of the transcripts.

Themes identified in this first stage were not specified *a priori*, but rather were derived from the data. Sections of text were coded by issue or theme; additional codes were added as new themes emerged. Themes and categories were then examined across the whole dataset and in relation to each specific interview. The research team then met to discuss and further refine each set of themes. Discrepancies were resolved by first revisiting and reviewing the data, and then through group discussion. Key themes were summarized, reviewed, and agreed upon by the study team members.

## Results

Among the 30 participants, 18 (60%) had not initiated the vaccine series; eight (26.7%) had received the first; and four (13.3%) had received all three HPV vaccine doses (Table 1). Of the seven participants born abroad, four had resided in the United States for fewer than 10 years. Unvaccinated young women were asked their reasons for not initiating the HPV vaccine series (*n*=18). The results presented here are organized around four major themes identified in the analysis: (1) lack of physician recommendation, (2) negative vaccine perceptions, (3) negative vaccine attitudes, and (4) side effect concerns (Table 2).

**Table 1.** **Unvaccinated Participants' Demographic Profile**

Characteristics Self-identity	n	Percent
Haitian	6	33
Haitian American	12	67
Class standing		
College graduate	2	11.2
Junior/senior	8	44.4
Freshman/sophomore	8	44.4
Country of birth		
USA	14	78
Haiti	4	22

**Table 2.** **Themes and Supporting Quotes from Unvaccinated Participants**

Theme	Supporting quotes
**Lack of physician recommendation**	
	You know, a lot of doctors do not talk to you about it. If I go to doctors for a routine checkup, they would not mention it. I thought it wasn't important since the vaccine was not mentioned or offered.
	When I asked her [my doctor] about it she told me that it would be up to me. And at the time I was not 18 yet, so my parents would have to sign for me to get it because it is not mandatory here nor at the school I go to. I guess that is why she didn't say nothing about it and never said nothing about it.
	I have not been offered the vaccine by a physician.
	The doctor did not recommend it.
	I don't recall.
	I can't remember, I have no idea; I don't even remember the last time I went to the doctor with my mom. Those are specific details that I can't really match; it's too far back.
	… I think she might have said it, but I don't remember.
	To be honest, I am going to say no, I don't recall. I really did not know much information about it. And I felt if this is something that I need…. If I'm coming to the doctor, they will bring it up to me….So if they don't bring it up to me, I probably don't need it.
**Negative vaccine perception**	
	As soon as it said you had to be….. It is something that is transmitted through sexual activity…. I was like well, I am not sexually active so that does not concern me. And I dismissed it.
	For safety precaution, if you are going to be sexually active, you might as well get it [the vaccine].
	When I read the paper, it said for sexually active people. And I am not sexually active.
	If you are thinking about becoming sexually active, then that is the best time to take it.
	I may take it, but I am not sexually active right now…So I don't see the need for it.
	Well, I'm not going to say I don't need it. I haven't taken it because I'm not sexually active.
	The best way you can prevent sexually transmitted diseases is to be smart about who you are with. The decisions that I have made… I don't sleep around; I always use protection. I take care of my health, so those are the methods I decide to use as opposed to taking medicine to fix something I could prevent.
	Anyone that's having sex I guess, or unprotected sex.
	It is not one of the vaccines I had to take for school.
	Because it was never mandatory, so that means it is not serious.
	I did not think it was necessary. I could've gotten it, but it was not a requirement.
**Negative vaccine attitudes**	
	Me personally, I'm not big on vaccines.
	My first instinct is not to get vaccines.
	Personally, I don't like it [needle]. I don't like shots. I don't like needles and anything like that. As far as I can, I stay away from it.
	Just because of the way I feel about the whole vaccine situation…. My fear of vaccines, as far as needles and everything like that ….and I don't feel like I'm in big need of it.
	Okay, it [HPV] exists. I acknowledge that, but it is not important to me personally…. The way they have advertised it and the limited information did not convince me.
**Side effect concerns**	
	I always have concerns regarding vaccines.
	Have you ever watched the commercials? It is like “Get this pill for that headache, but then you might feel dizzy; you might get stomachache; and your foot might fall off; and you may just die; but at least you will not have the headache anymore!”
	…. I had to take the flu shot. It made me extremely sick. So if I have to take another shot, I would probably say no because I was extremely sick for 3 or 4 days. It would influence my decision more than anything else if I had to take another vaccine.
	Sometimes you're given the vaccine, and in reaction to the vaccine, you actually get the infection. Certain things like that make me uneasy, so I'm not too sure.
	You have commercials about if you have taken this and this…. Then there is a lawsuit. And then you wonder “Are you really safe?” As much as you see commercials that this will prevent this, then you have commercials that if you have taken this shot or suffered this [adverse effect] from taking this medication…. Then it's too much.
	Because there's a lot of vaccines that my mom does not agree with, like the flu vaccine. And there is another one that she does not agree with just because of side effects.
	Just the side effects of it, which I don't know.

HPV, human papillomavirus.

### Lack of physician recommendation

Of the 18 participants who had not initiated the vaccine series, nine stated that they had not received a provider recommendation for the vaccine (*n*=9) (Table 2).

I remember one encounter with a physician…. He did not ask me. The initial question is always “Are you sexually active?” And I am like “no.” It would stop there. They would never ask me “Have you heard of this or would you like to get that?”

A few stated that they could not recall their healthcare provider recommending the vaccine (*n*=5). One young woman cited the healthcare providers' recommendations for several vaccines as her reason for not remembering the recommendation for HPV specifically.

I'm not sure; they offer a lot of vaccines. I do not remember if HPV is one of them.

### Negative vaccine perception

Among unvaccinated participants (*n*=18), the majority perceived HPV vaccine as a preventive measure for sexually active individuals (*n*=10). Five participants pointed out that they did not take the vaccine because (in their opinion) the vaccine is for sexually active women and they were not sexually active (Table 2).

It's a vaccine for cervical cancer. You should get the vaccine if you are sexually active.

A few of the 18 unvaccinated young women viewed HPV vaccine as a preventive measure for the sexually promiscuous and those who are having unprotected sex (*n*=3).

I do believe that this is a vaccine young women should get. Especially if she knows that she is being sexually active, and she has been around.

A few of those participants mentioned that the vaccine is not something they felt like they needed (*n*=3) (Table 2). A few participants mentioned the fact that the vaccine was not mandatory for school attendance. Hence, they did not feel a sense of urgency to get the vaccine (*n*=4).

Basically, I felt like that wasn't something I needed.

…The fact that it [the vaccine] is not an urgency, but it is an option. It's not like when you are growing up and you have to take shots before school. So, if it is an option, why take it? It is not like someone is telling you this is beneficial.

### Negative vaccine attitudes

Some participants expressed negative attitudes toward vaccines in general or the HPV vaccine (*n*=7). Two participants stated that they simply do not like vaccines, while three of those participants voiced their dislike for both vaccines and needles (Table 2).

I don't like shots so every time I hear about a vaccine, I never get excited to say “Oh I'm going to get a flu shot.” Even if it is free, I'm not going to go… I'm just not a fan.

### Side effect concerns

Some participants have also expressed side effect concerns regarding vaccines and medications in general (*n*=8). A few participants who commented on side effect concerns mentioned their personal disclaimer for vaccines in general (*n*=3). The remainder of those who had side effect concerns provided various rationales for their concerns (*n*=5) (Table 2).

I don't know anyone who has taken the vaccine. I don't know. It's a medication, and nowadays medications come with side effects.

Newness of the vaccine has been cited among the reasons for nonvaccination (*n*=3). One participant stated that her mother felt it was necessary to wait and see how individuals who have taken the vaccine react to it.

“Let's see how everybody else reacts to it.” She [my mother] was like “It is so new. You don't know what happens after people take it….”

## Discussion

Although the HPV vaccine has been available for about 10 years, several factors hinder vaccine uptake among Haitian American college women. Most of the unvaccinated participants have cited lack of physician recommendation as their reason for not receiving the vaccine. These findings reinforce previous studies that found lack of provider recommendation to be a barrier to vaccination.^27,33,36^

Although a physician recommendation does not always result in immediate vaccine uptake, it is a very important factor in medical decision-making among Haitians. Specifically, Haitian mothers highly value physician recommendations when making medical decisions.^44^ Study results indicate that Haitian American college women also value physician recommendations in their HPV vaccine decision-making. Thus, the lack of recommendation undermines the importance of the vaccine.

A few participants have mentioned that their healthcare provider would initially ask about their sexual activity status, and once they said they were not sexually active, the conversation would end there. Such interactions left participants with the impression that the vaccine was intended for young women who were sexually active. Providing a recommendation for the vaccine in this context is discordant with the guidelines as the vaccine is most effective before sexual debut. Moreover, because sexual abstinence is the cultural expectation for young, unmarried Haitian women,^44,47^ a vaccine recommendation in the context of sexual activity is culturally inappropriate and will likely result in vaccine refusal.

Unvaccinated participants have articulated their perception of the vaccine as a sexually transmitted infection preventive measure for sexually active women, women with multiple sex partners, and those who have unprotected sexual intercourse. Such HPV vaccine perceptions deter not only young women who are not sexually active or do not have multiple sex partners but also those who may be interested in the vaccine but are afraid of the stigma associated with sexually transmitted infections.

Unvaccinated participants have expressed negative vaccine attitudes. Several studies have reported a correlation between positive vaccine attitudes and HPV vaccine uptake.^21,44,48^ In the present study, participants who held negative vaccine perceptions felt that the HPV vaccine was a preventive measure they needed.

Some study participants voiced side effect concerns regarding the HPV vaccine. They mentioned that compared with the required vaccines, HPV vaccine has not been licensed long enough for them to be assured of its safety. A few participants mentioned that their mothers had side effect concerns because HPV vaccine was new compared with the vaccines required for school attendance. This finding lends support to results reported by Kobetz et al. ^9^ who found adult Haitian women to have fear of HPV vaccine due to its newness. In adolescents' and young adults' health decision-making, mothers play a central role.

Several young women commented on their mothers' interaction with the provider and their mothers' view regarding the vaccine. Participants' comments lend support to previous studies that have found mothers to be a central parental figure in HPV vaccine decision-making.^44,49^ Moreover, some participants have also mentioned adverse effects that were based on television commercials prompting individuals who have taken certain medications to contact law firms for potential compensation. Concerns about the potential adverse effects of the vaccine have been cited among factors that constitute an obstacle to vaccination.^22,32,50^

## Limitations and Strengths

This study focused on Haitian American college women in a region where there is a large Haitian population. As students at a large state university, they have access to a full university health center that includes a women's clinic. Hence, the study sample is not representative of college-aged Haitian women, particularly those not attending college. Additionally, some participants mentioned their sexual activity status at the time of the recommendation, but their sexual activity status at the time of their interview was not collected due to the sensitivity of this issue in Haitian culture. Therefore, participants could not be probed specifically about the influence of their sexual activity status on their decision to not be vaccinated.

Additionally, all interviews were conducted in English. Although all participants were enrolled in college, two had been living in the United States for fewer than 10 years. These participants could have been more comfortable expressing certain concepts and experiences in their native language; providing that option could have yielded a more detailed experience, especially from these two participants.

Unvaccinated participants were not specifically asked what could have facilitated them receiving the vaccine. Furthermore, this was an early inquiry study among college-enrolled women from one university. Hence, study results are not generalizable to all Haitian college women residing in the United States. Although the current study had several limitations, it sheds light on some of the barriers to HPV vaccination among Haitian American college women. Study results suggest that these women have misperceptions about the vaccine. These findings are important to facilitate HPV vaccine uptake in this population.

## Implications for Future Research and Practice

Study findings echo that lack of provider recommendation, side effect concerns, negative HPV vaccine perceptions, and attitudes constitute barriers to HPV vaccination among Haitian college women. Participants viewed HPV vaccine as new. Therefore, during medical visits, health practitioners should take the opportunity to not only make the recommendation to unvaccinated individuals but also to inform these patients that the vaccine has been licensed since 2006 and that its side effects are similar to those of required vaccines.

Participants also perceived HPV vaccine as a preventive measure for a sexually transmitted infection. It is important that providers refrain from asking participants about their sexual activity when recommending the vaccine as that may reinforce the misconception that the vaccine is intended for sexually active or even promiscuous individuals. HPV vaccine is directed at the prevention of HPV-related cancers. Hence, it is vital that providers emphasize that HPV is a cancer prevention vaccine not only for these women who are at high risk of cervical cancer but also for all eligible patients. Moreover, sexual abstinence is expected of young Haitian women.

Given that cultural expectation, providers should accentuate the fact that the vaccine is best taken before HPV exposure and that the vaccine confers less protection to sexually active individuals. Such accentuation can positively influence their HPV vaccine perception and would be culturally appropriate. Future research should investigate HPV vaccine uptake among Haitian adolescent boys and young men as HPV vaccine is recommended for both boys and girls.

In light of the high burden of cervical cancer among Haitian women, the few studies addressing HPV vaccine uptake in the Haitian population are conducted among women. However, HPV-related morbidity affects both men and women. Therefore, studies examining HPV vaccine uptake among adolescent boys and young men from ethnic groups disproportionately affected by HPV-related morbidity are needed.

## Conclusions

College women in this ethnic group face several barriers to HPV vaccination. Study findings are indicative that Haitian American college women have negative perceptions and attitudes that constitute obstacles to vaccination. Despite the negative perceptions and attitudes and side effect concerns among unvaccinated participants, Haitian American college women highly value provider recommendations for medical decision-making. Their high esteem of provider recommendations provides an opportunity for education that will dispel HPV vaccine myths and help engender positive HPV vaccine perceptions. It is important for providers to be mindful of the positive influence of their recommendation on women who heavily rely on it to accept or at least consider the HPV vaccine.

## Abbreviation Used

HPV: human papillomavirus
